# Genotype Diversity and Spread of *White Spot Syndrome Virus* (WSSV) in Madagascar (2012–2016)

**DOI:** 10.3390/v13091713

**Published:** 2021-08-28

**Authors:** Alain Moïse Onihary, Iony Manitra Razanajatovo, Lydia Rabetafika, Alexandra Bastaraud, Jean-Michel Heraud, Voahangy Rasolofo

**Affiliations:** 1 Food and Environmental Hygiene Laboratory, Institut Pasteur de Madagascar, Antananarivo 101, Madagascar; moise@pasteur.mg (A.M.O.); abastaraud@pasteur.mg (A.B.); 2 Animal Biology Department, Faculty of Sciences, University of Antananarivo, Antananarivo 101, Madagascar; rabetafikalaurence@gmail.com; 3 Ecole Doctorale Science de la Vie et de l’Environnement, Faculty of Sciences, University of Antananarivo, Antananarivo 101, Madagascar; ionyr@pasteur.mg; 4 Virology Unit, Institut Pasteur de Madagascar, Antananarivo 101, Madagascar; 5 Virology Department, Fondation Institut Pasteur de Dakar, Dakar 12900, Senegal; 6 Scientific Direction, Institut Pasteur de Madagascar, Antananarivo 101, Madagascar

**Keywords:** *White Spot Syndrome Virus* (WSSV), aquaculture, Madagascar, genotype, virology

## Abstract

White Spot Disease (WSD) caused by the *White Spot Syndrome Virus* (WSSV) is the most devastating viral disease threatening the shrimp culture industry worldwide, including Madagascar. WDS was first reported on the island in 2012; however, little is known about the circulation of the virus and its genetic diversity. Our study aimed at describing the molecular diversity and the spread of WSSV in the populations of Madagascan crustaceans. Farmed and wild shrimps were collected from various locations in Madagascar from 2012 to 2016 and were tested for WSSV. Amplicons from positive specimens targeting five molecular markers (ORF75, ORF94, ORF125, VR14/15 and VR23/24) were sequenced for genotyping characterizations. Four genotypes were found in Madagascar. The type-I genotype was observed in the south-west of Madagascar in April 2012, causing a disastrous epidemic, then spread to the North-West coast. Type-II strains were detected in October 2012 causing an outbreak in another *Penaeus monodon* farm. In 2014 and 2015, types II and III were observed in shrimp farms. Finally, in 2016, types II and IV were found in wild species including *Fenneropenaeus indicus, Metapenaeus monoceros, Marsupenaeus japonicus* and *Macrobrachium rosenbergii*. Considering the economic importance of the shrimp industry for Madagascar, our study highlights the need to maintain WSSV surveillance to quickly take appropriate countermeasures in case of outbreak and to sustain this industry.

## 1. Introduction

*White Spot Syndrome Virus* (WSSV) is the only virus belonging to the genus Whispovirus within the Nimaviridae family [1]. WSSV is the causative agent of White Spot Disease (WSD) and it is the most significant viral pathogen of cultured shrimp [2]. This virus has a wide range of potential hosts [3], including lobsters, crayfish and crabs [3,4]. For instance, WSSV infection was found in a variety of marine crabs and spiny lobsters without significant loss or viral disease (WSSV carriers) [3,4,5,6]. However, a more recent study reported WSSV infection with clinical signs in some species of crabs and lobsters [7,8,9]. The clinical signs of the disease include lethargy, anorexia, white spots on the cuticle and, often, generalized reddish to pink discoloration [10]. This virus is highly contagious, and transmission can occur either horizontally through oral ingestion of infected prawns or contaminated water, or vertically from infected parents in hatcheries [11].

Since its first occurrence in China and Taiwan during 1991 to 1992 [12], the virus has spread rapidly through Asia [3,13], the Americas [14], Middle East, Africa [15,16] and Australia [17,18]. In cultured shrimps, WSSV infection can lead to a loss of up to 100% within 1–10 days [19]. This is the most serious problem threatening the shrimp culture industry worldwide [2]. Economic losses due to this disease have been estimated at up to US $15 billion, since its emergence in 1991, increasing at a rate of US $1 billion annually [20,21]. Although these estimates are dated from more than 10 years ago, more recent data reported that the global shrimp sector lost US $6 billion in 2016 due to viral diseases [22].

Madagascar is the fourth biggest island in the world with a total surface area of 587,295 km^2^ and is located in the south-west of the Indian Ocean. Shrimp farming was introduced in Madagascar in the early 1980s and most farms are located on the north-west coast of the country, along the Mozambican channel. Shrimp (captured and farmed) is the most valuable fishery in the country. Commercial shrimp farming represents an important resource for the economy of the country and provides many jobs [23]. In 1998, 16,000 MT (6000 MT from shrimp farming) of shrimps were produced in Madagascar, with a total value estimated at US $54 million [24,25]. In 2012, production dropped to 4940 MT [25], not reaching the production levels of previous years. Interestingly, the first cases of WSD were reported in April 2012 in a farm located on the south-west coast of Madagascar [16]. This decrease in production could be attributed to the occurrence of WSD outbreak [25]. A few months later (September 2012), WSD was again detected in another shrimp farm located on the west coast. Furthermore, more farms located on the same axis were affected by WSD between 2012 and 2016. A previous study by Tang et al. [16] investigating the potential origins of the WSSV from the first farms affected by WSD in 2012 revealed that the WSSV found in Madagascar, Mozambique and Saudi Arabia were from a common source, probably the Indian Ocean and the Red Sea. To further explore the origins of the WSSV found in Madagascar, we conducted a study aiming at characterizing the genotypes of WSSV that circulated amongst cultured and wild shrimps between 2012 and 2016. This is the first report looking at the genetic diversity of WSSV present in commercial farms and wild shrimps in Madagascar.

## 2. Materials and Methods

### 2.1. Specimen Collections

**Figure 1:**
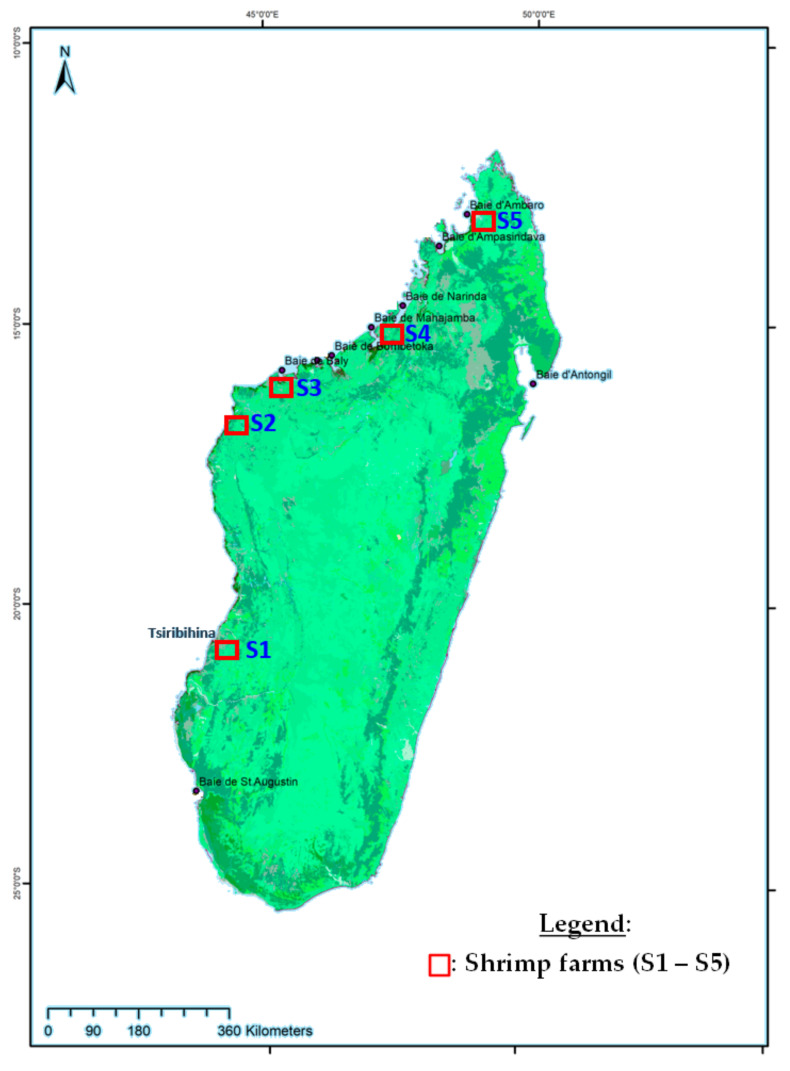
Sampling sites for crustaceans during the study. The Shrimp farms are located in Belo sur Tsiribihina (**S1**), Sambao estuary (**S2**), Baly bay (**S3**), Mahajamba bay (**S4**) and Ambaro bay (**S5**).

Cultured *Penaeus monodon* and bycatch of *Fenneropenaeus indicus* were collected from five shrimp farms (Figure 1: S1 to S5). Wild crustaceans were collected from the mangrove swamps downstream of four shrimp farms (Figure 1: S1 to S5) and near the first farm infected, located in the district of Belo sur Tsiribihina (Figure 1: S1). The sampling was carried out from April 2012 to December 2016. Each sample consisted of whole juvenile or adult shrimps. All of them were preserved in 96% ethanol or by freezing until analysis at the Food and Environmental Hygiene Laboratory of the Institut Pasteur de Madagascar.

### 2.2. Amplification and Detection of WSSV

Total DNA was extracted from 25 mg of shrimp pleopod using a Nucleospin^®^ tissue Kit (Macherey-Nagel, Dueren, Germany) according to the manufacturer’s protocol.

Screening and detection of WSSV were conducted according to standard protocols (Table 1) as described previously [13], with some modifications. The nested PCR was sensitive and allowed us to detect a light WSSV infection in wild shrimps or in cultured shrimps outside an epidemic period. The first-step PCR used 146F1 and 146R1 primers with an expected amplified fragment size of 1447 bp (Table 1) in the SalI DNA fragment of the WSSV genome. The PCR reaction of the first step was carried out with 20 µL of reaction mixture that consisted of 1 U of Platinum Taq DNA polymerase (Invitrogen, Waltham, MA, USA), 1× PCR buffer (10 mM Tris–HCl, KCl 50 mM, pH 9), 1.5 mM of MgCl_2_, 0.5 µM of each primer, 0.2 mM of dNTPs and 4 µL of template DNA at 50 ng µL^−1^ and made up to volume using PCR grade distilled water.

Primers 146F2 and 146R2 (Table 1) were used for nested (2 step) PCR to amplify a 941 bp DNA fragment inside the first strand of 1447 bp in the SalI. The nested PCR included 10 µL of the first step reaction mixture, 1.25 U of Taq DNA Polymerase, 1× PCR buffer, 1.5 mM of MgCl_2_, 1 µM of each primer, 0.2 mM of dNTPs and made up to 25 µL using PCR grade distilled water.

Amplification was performed in a DNA Thermocycler (Biometra T-Gradient, Biometra, Gottingen, Germany). The first step was cycled using the following parameters: initial denaturation at 94 °C for 4 min, 55 °C for 1 min, 72 °C for 2 min followed by 40 cycles of 94 °C for 1 min, 55 °C for 1 min, 72 °C for 2 min, with a final extension at 72 °C for 5 min. For the nested-PCR, the following conditions were used: 40 cycles of 94 °C for 1 min, 62 °C for 1 min, 72 °C for 2 min and a final extension at 72 °C for 5 min. The PCR product (10 µL) was mixed with 2 µL of loading dye buffer and subjected to electrophoresis in 1.2% of agarose gel, containing 5% of ethidium bromide. Fragment sizes were determined using a 100 bp DNA Ladder (GeneRuler, Thermo Scientific, Paisley, UK) and the gels were observed and photographed under UV light using Visioncapt (Transluminator, Biovision, Bengaluru, Karnataka, India).

The DNA extracted from WSSV infected shrimp tissue from the University of Arizona was used as positive control. DNA from a WSSV negative field sample and sterile distilled water was used as the negative control.

### 2.3. WSSV Genotyping

WSSV-positive samples were amplified using three VNTR loci (ORF75, ORF94, and ORF125) and two variable regions (VR14/15, VR23/24). These five molecular markers have been used efficiently and widely for WSSV genotyping [26,27]. PCR reactions were performed in a final volume of 25 µL, containing 1.25 U of Platinum Taq DNA polymerase (Invitrogen, Waltham, MA, USA), 1× PCR buffer (10 mM Tris–HCl, KCl 50 mM, pH 9), 1.5 mM of MgCl_2_, 0.5 µM of each primer, 0.2 mM of dNTPs, 50 ng µL^−1^ of extracted DNA in 2.5 µL volume and adjusted to 25 µL with PCR grade distilled water. Amplification was performed according to previously described primers, with some modifications. We hypothesize that if no PCR products were detected, the targeting DNA fragment is missing in the WSSV genomic sequence. To ensure that the gene is deleted, we performed a new PCR with another set of primers, flanking the targeted genomic DNA sequence (Table 1).

### 2.4. DNA Sequencing Analysis

The PCR-amplified products were sequenced by “Genoscreen Services” for forward and reverse Sanger sequencing with the same primers used in the PCR reaction. Nucleotides sequences were edited and aligned using BioEdit Sequence Alignment Editor Software V7.1 program [28]. Consensus sequences were blasted to available genes and whole genome of WSSV downloaded from the NCBI website. The WSSV sequence of Thailand strain TH-96-II (GenBank Accession AY753327) was used as a reference for variable region VR14/15 analysis [29] and WSSV sequence of Taiwan strain WSSV-TW (GenBank Accession AF440570) was used for variable region VR23/24 [30]. The deletion sizes of these variable regions were then compared to WSSV strains described. The numbers of tandem repeats for VNTRs loci (ORF75, ORF94, ORF125) were analyzed using the Tandem Repeats Finder (TRF) program [31]. The same sequences of repeat fragments as described previously [15,16,29,32,33,34] were used to describe WSSV strains: 45 bp and 102 bp repeat units (RUs) for ORF75, 54 bp RU for ORF94 and 69 bp RU for ORF125. The WSSV genotype was characterized as “N75, N94, N125, X14/15, X23/24” where N is the number of repeat units in a specific ORF and X is the number of base pair deletions in the variable region VR14/15, VR23/24.

## 3. Results

From April 2012 to December 2016, a total of 2184 specimens were collected and tested for the presence of WSSV. Among them, 503 were positive. These positive specimens were collected from five shrimp farms (Figure 1: S1 to S5) from 2012 to 2015 and in three mangroves swamps (Narinda bay, Mahajamba bay, Tsiribihina estuary) from 2014 to 2016.

### 3.1. WSSV Screening and Genotyping

We first screened the 503 positive samples with the ORF125 locus for genotyping. Of these, 308 samples gave four distinct PCR product sizes for this VNTR region (data not shown). From these 308 samples, samples with moderate to heavy infection (positive result in the first step PCR) were selected for further analysis. Forty (40) were then used for full genotyping with the five molecular markers. These samples were chosen according to their origin (cultured or wild shrimps), the sampling date and the host species. Of these 40 samples, 18 were from farmed shrimps and 22 from the wildlife. Sampling included 11 samples of *Penaeus monodon*, 21 of *Fenneropenaeus indicus*, 5 of *Metapenaeus monoceros*, 1 of *Marsupenaeus japonicus* and 2 of *Macrobrachium rosenbergii* (Table 2).

### 3.2. VNTR Analysis of ORF125

Four amplicons of 583 bp, 652 bp, 721 bp, and 792 bp (GenBank Accession MZ327619 to MZ327622, Supplementary Table S1) were generated with the amplification of ORF125. Sequences alignment and TRF program analysis of these amplicons showed 5, 6, 7 and 8 repeat units of 69 bp fragments for 583 bp, 652 bp, 721 bp and 792 bp respectively. The number of samples showing these amplicon sizes was 5, 27, 4 and 4 respectively (Table 2).

### 3.3. VNTR Analysis of ORF94

No PCR products were obtained using the primer ORF94-F and ORF94-R for all samples. A second PCR amplification targeting ORF93 and ORF96 genes was done using the couple of primers ORF93-F1/ORF96-R1 used by Tang et al. in 2013 [16] (Table 1). An amplicon of 348 bp (GenBank Accession MZ327623, Supplementary Table S1) was generated for the analysis of all forty samples. Sequencing and alignment using BLAST of this amplicon showed 99% sequence identity with other WSSV sequences (WSSV-CN04, GenBank Accession KY827813; WSSV-TH, GenBank Accession AF369029 and WSSV-TW, GenBank Accession AF440570). Sequence analysis demonstrated that the 348 bp sequence matched with the sequence of ORF93 and ORF96 in these reference genomes. Thus, ORF94 and ORF95 were deleted for all Malagasy WSSV strains (Table 2).

### 3.4. VNTR Analysis in ORF75

PCR amplification of ORF75 with primers ORF75-flank-F/ORF75-flank-R [26] failed to produce PCR amplicons for all samples. Second amplification targeting ORF73 and ORF77 genes was done using the couple of primers ORF73-F and ORF77-R [16]. An amplicons of 1739 bp (GenBank Accession MZ327624, Supplementary Table S1) were generated for 35 samples. No PCR product was detected for 5 samples. These same results were observed with a third PCR amplification using the primer set TJW75 as described by Piamsomboon et al. in 2018 [34] (Table 1). The 35 samples described above gave 702 bp amplicons and no PCR product for the five remaining samples.

Sequencing of the 1739 bp amplicon and BLAST (Basic Local Alignment Sequences Tools) with WSSV DNA sequences available online showed 100% sequence identity with WSSV-CN04 (GenBank Accession KY827813). DNA sequence analysis using TRF program allowed us to confirm that 3 RUs of 45 bp and 102 bp were observed in these DNA sequences: two 45 bp RU and one 102 bp RU (Table 2).

### 3.5. Deletion in VR14/15

**Figure 2:**
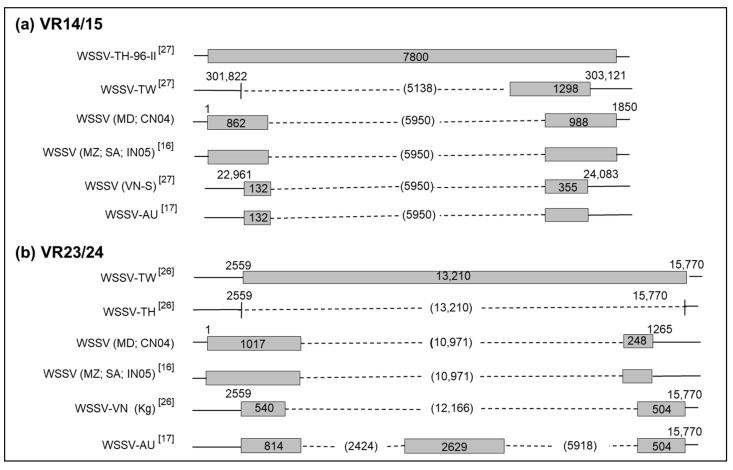
Schematic representation of the variable regions VR14/15 (**a**) and VR23/24 (**b**). Fragment lengths (bp) are described in boxes. Nucleotide position numbers in 5′ and 3′ regions are indicated above each strain according to GenBank sequences. Dashed lines indicate the deleted sequences. Deletion size is given in brackets. MD: Madagascar, MZ: Mozambique, SA: Saudi-Arabia, TH: Thaïland, TW: Taïwan, CN: China, VN: Vietnam, IN: India, AU: Australia (GenBank Accession MF768985).

The first PCR using the primer set ORF14/15-complete [29] gave 1850 bp amplicon (GenBank Accession MZ327625, Supplementary Table S1) for 20 samples out of the 40 samples. To investigate the reason for these negatives results, the two-step PCR as described previously by Piamsomboon et al. in 2018 [34] was applied to the samples. The first step used the primer set TJW14/15 and the second step used the primer set VR14/15-screen (Table 2). Amplicon sizes of 600 bp were detected in 35 of 40 samples. The last five samples failed to produce PCR amplicons. Sequencing and alignment of the 1850 bp amplicon using BLAST gave 100% identity with WSSV-CN04 (GenBank Accession KY827813) and more than 99% with other WSSV sequences (WSSV-TH, GenBank Accession AF369029; WSSV-TW, GenBank Accession AF440570). Compared to WSSV-TH-96-II reference sequence of 7800 bp (GenBank Accession AY753327), the first 862 bp are located in the 5′ region and the remaining 988 bp are located in the 3′ region of the WSSV-TH-96-II. This indicated that Malagasy WSSV strains are characterized by a 5950 bp deletion size (Table 2, Figure 2).

### 3.6. Deletion in VR23/24

The primer set VR23/24-south [26] generated a PCR amplicon of 1265 bp (GenBank Accession MZ327626, Supplementary Table S1) for 26 of 40 samples. Other PCR amplifications were achieved for 14 out of 40 samples using the primers as described by Dieu et al. in 2004, Jiang et al. in 2017, and Piamsomboon et al. in 2018 [26,34,35] (Table 1). None of these PCR amplifications was unable to produce an amplicon for the 14 remaining samples. DNA sequence and alignment of the 1265 bp amplicon with BLAST showed 100% identity with WSSV-CN04 (GenBank Accession KY827813). Compared to WSSV-TW (GenBank Accession AF440570), the 1017 bp fragment is located in the 5′ region and 248 bp were located in the 3′ region of the WSSV-TW sequence. This means that 10,971 bp were deleted for all Malagasy WSSV strains (Table 2, Figure 2).

### 3.7. WSSV Genotype and Its Circulation

**Figure 3:**
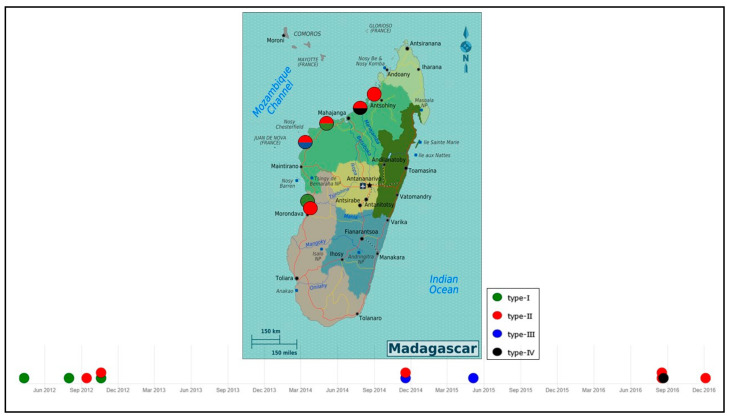
Genotypes of WSSV in the West coast of Madagascar from 2012 to 2016. WSSV genotypes are designed as type-I, type-II, type-III and type-IV (Simulation available at: https://microreact.org/project/eSrC2wJQrXgcxpKZvT4auZ/8432e8b4, accessed on 18 May 2021).

Four WSSV genotypes were detected after molecular analysis of 40 different shrimp samples. The same genotype nomenclature as previously described was used in this study [16]. Each of them was designated as type I ({7_125_, del_94_, 3_75_, ∆5950_14/15_, ∆10971_23/24_}), type II ({6_125_, del_94_, 3_75_, ∆5950_14/15_, ∆10971_23/24_}), type III ({5_125_, del_94_, 3_75_, ∆5950_14/15_, ∆10971_23/24_}), and type IV ({8_125_, del_94_, 3_75_, ∆5950_14/15_, ∆10971_23/24_}). These four genotypes differed in the number of 69 bp RU in the ORF125 gene. Type II with 6 RU (ORF125-6) was observed in 27 of 40 samples and was detected in cultured as well as in wild shrimp species collected from 2012 to 2016. The three other WSSV variants, type I (ORF125-7), type III (ORF125-5) and type IV (ORF125-8), were detected sporadically in cultured or in wild shrimp species from 2012 to 2016. The first WSD outbreak in April 2012 was recorded in the south-west of Madagascar (S1) and was due to WSSV strain type I. WSSV type II was observed in September 2012 within S2 and S3 (Figure 2). In 2014 and 2015, type III appeared and was detected in four samples of *P. monodon* and *F. indicus* collected in S2 farm at the same time as type II. In 2016, type IV was detected in some wild *F. indicus* samples collected along the estuary of Tsiribihina, near S1 on the southern-west coast of Madagascar. Genotypes of WSSV on the west coast of Madagascar from 2012 to 2016 were mapped and simulated using microreact [36] (Figure 3). This microreact project can be visualized by sites of infection, by WSSV-genotypes or by host species (https://microreact.org/project/eSrC2wJQrXgcxpKZvT4auZ/8432e8b4, accessed on 18 May 2021).

## 4. Discussion

Madagascar was free of WSSV until April 2012, according to the national WSSV surveillance plan conducted by the Malagasy authorities, in the wild and at all aquaculture sites. Since then, several species of wild and farmed shrimp have been found to carry WSSV. According to Tang et al. [16], the first genotype named type I ({7_125_, del_94_, 3_75_, ∆5950_14/15_, ∆10971_23/24_) was detected in the south-west of the country in April 2012, followed by type II ({6_125_, del_94_, 3_75_, ∆5950_14/15_, ∆10971_23/24_}) in September 2012, in one farm closest to the first infected farm. In 2014 and 2015, WSSV type II and type III ({5_125_, del_94_, 3_75_, ∆5950_14/15_, ∆10971_23/24_}) were observed in other farms located further north of the previously infected farms. In 2016, type II and type IV ({8_125_, del_94_, 3_75_, ∆5950_14/15_, ∆10971_23/24_}) were found in wild shrimp species from Mahajamba bay, Narinda bay and the estuary of Tsiribihina. These genotypes and mainly WSSV type II seemed to have spread progressively from the south to the north of the west coast of Madagascar and reached the mangrove areas. This pattern is similar to the Indian Ocean current that reaches Madagascar on the east coast and then from the south moves from south to north [37], related to the movement of crustaceans. Once the virus was present in the wild [16], the capture of spawners, the presence of healthy carriers in ponds (crabs or certain of species of barnacles attached to the gill of wild mud crab, lobsters, copepods and insect larvae) [38,39], or the probable use of contaminated water by pumping seawater, could explain the introduction of this virus into aquaculture sites. Certain species of seabirds observed around all farms in Madagascar have also been reported as potential sources of virus transmission [11]. In addition, the proximity of the shrimp farm to the sea is correlated with WSSV infection [40]. This could explain why no outbreaks of WSSV have been reported in the only shrimp farm (Figure 1: S5) located on the extreme north of Madagascar. These results suggest an evolution and local spread of WSSV on the west coast of Madagascar between 2012 and 2016.

In this study, two new genotypes are reported for the first time in Madagascar. These are WSSV type III ({5_125_, del_94_, 3_75_, ∆5950_14/15_, ∆10971_23/24_}) and type IV ({8_125_, del_94_, 3_75_, ∆5950_14/15_, ∆10971_23/24_}). While genotypes I and II were similar to those previously reported in Mozambique or Saudi Arabia, the types III and IV described here are different from the genotypes detected in Saudi Arabia ({8_125_, 13_94_, 3_75_, ∆5950_14/15_, ∆10971_23/24_}) and ({6_125_, 7_94_, 3_75_, ∆5950_14/15_, ∆10971_23/24_}) [15,16]. Arabian strains of WSSV had 13 and 7 RUs of 54 bp in the ORF94 sequence, whereas the Malagasy strains had a complete deletion of ORF94. However, ORF125-8 and ORF125-6, with the same ORF75-3, ∆5950_14/15_, ∆10971_23/24_, were indeed previously reported in Saudi Arabia (2011) [15]. These results reinforce the hypothesis that the WSSV strains observed in Madagascar, Mozambique, and Saudi Arabia may have a common evolutionary origin.

We also found that the Malagasy strains (WSSV-MD) were genetically very similar to the Chinese strain (WSSV-CN04, Accession GenBank KY827813). The WSSV-CN04 had an identical deletion of 5950 bp at VR14/15 and 10,971 bp at VR23/24 similarly to the Malagasy strains. Furthermore, the Australian (WSSV-AU, GenBank Accession MF768985) and Vietnamese (WSSV-VN-S) strains also carried a similar deletion size of 5950 bp at VR14/15 compared to Malagasy strains. [17]. In addition, Malagasy and Australian strains shared the same RUs on VNTRs regions ({7_125_, del_94_, 3_75_}). The variable regions (VR14/15 and VR23/24) and VNTR are used for global and regional molecular epidemiology, respectively [27,32]. These results suggest that the strains observed in Madagascar might share a common lineage with WSSV from China or Australia. In contrast, WSSV-CN04 had different RUs at VNTRs regions ({4_94_, 6_75_}) compared to Malagasy strains ({del_94_, 3_75_}) and WSSV-AU showed two deletions of 2414 bp and 5918 bp at VR23/24 while WSSV-MD had a 10,971 bp deletion at this same locus. Comparative analysis of the whole genome of WSSV showed that Chinese and Australian strains are clustered together [17]. However, this whole genome approach could be biased by the presence of the large deletions [41]. In addition, it has been shown that adaptation of the virus to environmental conditions or passage to different host species subsequently favors the selection of different genotypes [42]. Thus, the source of the virus in Madagascar remains unclear. An alternative genotyping marker, selected according to our results and those described previously [41], could be used to better understand the genetic diversity and the source of WSSV.

This study demonstrated the effective use of previously described primers for molecular epidemiology of WSSV. The analysis of VNTRs (ORF75, ORF94 and ORF125) can be applied in Madagascar following the recommendations from several authors [16,26,27,33]. Indeed, these markers are useful for local molecular epidemiology [32]. The use on sequences of VR14/15 and VR23/24 increases the sensitivity of the genotyping of WSSV by PCR [16,34]. Specimens collected in 2014 from the northern part of the Island and mangrove areas had lower viral loads compared to those obtained during epidemic periods and in the south and west. For these low viral load specimens, the use of a nested PCR allowed detection of the virus. VR14/15 amplification, initially negative with conventional PCR amplification on some samples, yielded positive results by amplifying first with the TJW14/15 (first step) primers followed by a second PCR targeting the VR14/15 region, as reported by Piamsomboon et al. [34]. More strains were characterized using these primers and the results will serve as a solid basis for future research and needs in aquaculture. However, despite the use of this primer panel, some samples remained negative (including five samples for ORF75 and VR14/15, and 14 samples for VR23/24) even with a viral load ranging from moderate to severe. Therefore, a different approach should be developed, either by designing more sensitive primers for the detection of Malagasy strains or by implementing unbiased amplification such as the next generation sequencing approach.

Our study has some limitations. Indeed, following the outbreaks that affected the countries in 2012, it was difficult to receive regular specimens from the wild as well as from some aquaculture sites. Some farmers decided to do their testing abroad and we did not have access to the results of these tests. Our observation covers the period between 2012 and 2016 and we cannot exclude that the current situation has changed in terms of distribution and genotypic circulation. Analyses of a larger number of WSSV sequences collected from different locations and time periods (epidemic and non-epidemic) would identify all circulating genotypes in Madagascar and provide more details on the distribution and frequency of each genotype as well as the potential virulence of the different genotypes. Further analysis using an alternative genotyping method [41], combined with an experimental virulence study of each strain, could clarify some of the conflicting opinions. Indeed, some authors argue that the reduction in genome size is correlated with the virulence and pathogenicity of the virus [43]. In contrast, others argue that smaller genomes are not necessarily associated with higher mortality [44].

## 5. Conclusions

This study revealed that at least four distinct strains of WSSV are circulating in Madagascar, both in the wild and in aquaculture. The WSSV type I strain was responsible for the first outbreak in the southwestern part of Madagascar in 2012. The virus apparently spread from southern to northwestern Madagascar. The type II genotype appeared a few months later and was responsible for further outbreaks in different localities of the west coast. Genotypes III and IV were present in breeding species of *P. monodon* or species caught incidentally in breeding ponds such as *F. indicus*. These genotypes were also detected in the environment in several wild species of crustaceans (*M. monoceros*, *M. japonicus*, *M. rosenbergii*). Our results support a common origin of WSSV from Madagascar, Mozambique and Saudi Arabia as described previously and also suggest that Malagasy strains share a common lineage with the Chinese (WSSV-CN04) and Australian (WSSV-AU) strains, based on the similarity of the number of RUs in VNTRs regions and the similar size of the deletions for China and Madagascar.

We conclude that these three VNTRs markers and two variable regions are useful for local and large-scale epidemiological study. However, additional study would be needed to describe which of these genotypes are involved in the white spot outbreaks recorded almost every year in Madagascar from 2012 to 2020, as well as their virulence levels. New primers more specific to the strains observed in Madagascar could be added to those already used to describe the current situation on the big island with this pathogen. The hypothesis of new infestation from the same source should not be ruled out. An alternative genotyping method including whole genome sequencing of representative strains should be carried out in Madagascar for a more accurate in-depth epidemiological study. Experimental trials would be interesting to better understand the correlation between virulence and genome size.

As no effective vaccine or treatment is currently available against WSSV-associated infection, the aquaculture industry must adapt and evolve by monitoring all parameters that favor WSSV outbreaks. Control measures are based on risk management combined with strict biosecurity measures and an adequate surveillance plan for early detection of the virus, to reduce the impact on production. Currently, a significant progress is observed in the selection of WSSV-resistant shrimp strains in aquaculture sites. Thus, this study would be a significant advance in the selection of shrimp resistant to WSSV strains circulating in Madagascar.

## Figures and Tables

**Table 1:** Primers used and cycling conditions for WSSV screening and variable loci analysis. (ORF = Open reading frame, VR = Variable region).

	Primers	Forward/Reverse Primers	Sequence (5’–3’)	Cycling Conditions	PCR Product Size (bp)	References
WSSV screening	146-1 (first step)	146-F1	ACTACTAACTTCAGCCTATCTAG	94 °C 4 min, 55 °C 1 min, 72 °C 2 min; 40 × [94 °C 4 min, 55 °C 1 min, 72 °C 4 min]; 72 °C 5 min	1447	[13]
		146-R1	TAATGCGGGTGTAATGTTCTTACGA			
	146-2 (second step)	146-F2	GTAACTGCCCCTTCCATCTCCA	40 × [94 °C 1 min, 62 °C 1 min, 72 °C 2 min]; 72 °C 5 min	941	
		146-R2	TACGGCAGCTGCTGCACCTTGT			
Variable loci First PCR	ORF75-flank	ORF75-flank-F	GAAGCAGTATCTCTAACAC	94 °C 4 min; 40 × [94 °C 1 min, 49/50 °C 80 s, 72 °C 1 min]; 72 °C 5 min	868	[26]
		ORF75-flank-R	CAACAGGTGCGTAAAAGAAG			
	ORF94	ORF94-F	TCTACTCGAGGAGGTGACGAC	94 °C 3 min; 35 × [94 °C 30 s, 55 °C 30 s, 72 °C 1 min]; 72 °C 7 min	506 to 1262	[33]
		ORF94-R	AGCAGGTGTGTACACATTTCATG			
	ORF125-flank	ORF125 flank-F	CGAAATCTTGATATGTTGTGC	94 °C 3 min; 35 × [94 °C 30 s, 55 °C 30 s, 72 °C 1 min]; 72 °C 7 min	652	[26]
		ORF125 flank-R	CCATATCCATTGCCCTTCTC			
	VR14/15-complete	VR14/15-complete-F	AATATGGAACGACGGGTG	94 °C 3; 35 × [94 °C 30 s, 50 °C 30 s, 72 °C 2 min]; 72 °C 7 min	1851	[29]
		VR14/15-complete-R	GACCAGCGCCTCTTCAG			
	VR23/24-south	VR23/24-south-F	GTAGTGCATGTTTCTCTAAC	94 °C 3 min; 35 × [94 °C 30 s, 45 °C 30 s, 72 °C 2 min]; 72 °C 7 min	1264	[27]
		VR23/24-south-R	GTAAGTTTATTGCTGAGAAG			
Variable loci Second PCR	ORF73/ORF77	ORF73-F	CTTTCACCGCTCTCACCAAC	94 °C 3 min; 35 × [94 °C 30 s, 55 °C 30 s, 72 °C 2 min]; 72 °C 7 min	1739	[16]
		ORF77-R	GGGTTCACCAGAGAGACAGG			
	ORF93/ORF96	ORF93-F1	CGCCCTATTACCATTGATGC	94 °C 4 min; 40 × [94 °C 1 min, 58 °C 60 min, 72 °C 1 min]; 72 °C 5 min	348	[15]
		ORF96-R1	GCAACAAATTCCCCTTTCAA			
	TJW14/15	TJW14/15-F	TCAACAACCCAAATCCCATT	94 °C 3 min; 40 × [94 °C 15 s, 60 °C 15 s, 72 °C 1 min]; 72 °C 5 min	3343	[34]
		TJW14/15-R	CTCTCAATCTTCCCCCAACA			
	VR14/15-screen	VR14/15-screen-F	GAGATGCGAACCACTAAAAG	94 °C 3 min; 40 × [94 °C 15 s, 60 °C 15 s, 72 °C 1 min]; 72 °C 5 min	600	[26]
		VR14/15-screen-R	ATGGAGGCGAGACTTGC			
	VR23/24-screen	VR23/24-screen-F	CACACTTGAAAAATACACCAG	94 °C 3 min; 40 × [94 °C 15 s, 49 °C 65 s, 72 °C 1 min]; 72 °C 5 min	548	
		VR23/24-screen-R	GTAAGTTTATTGCTGAGAAG			
Variable loci Third PCR	ORF75	TJW75-F	TCTGAAGCTGGGGGAACTAA	94 °C 3 min; 40 × [94 °C 15 s, 60 °C 15 s, 72 °C 1 min]; 72 °C 5 min	702	[34]
		TJW75-R	GAGCAACTCTGCACAGCATC			
	VR23/24-south04	VR23/24-south04-F	CTACAACGGCCAAGTCAT	94 °C 3 min; 40 × [94 °C 15 s, 60 °C 15 s, 72 °C 1 s]; 72 °C 5 min	1500/2000	[34]
	VR23/24-1	VR23/24-1-R	ATGATTGTATTCGTCGAAGG			
	ORF23/24	ORF23/24-F	GGTAGGAGAAGGTACGCACG	94 °C 3 min; 40 × [94 °C 30 s, 60 °C 15 s, 72 °C 1 min]; 72 °C 5 min	4025	[35]
		ORF23/24-R	GCCCAGATTGGTCATGTCCA			

**Table 2:** Study samples, number of repeat units (No of RUs) in VNTRs regions (ORF75, ORF94, ORF125) and deletion size in variable regions (VR14/15, VR23/24) of WSSV.

Site	Type	Host Species ^1^	Sampling Date	Nb. of Samples	Nb. of Repeat Units			Deletion Size (bp)	
					ORF75	ORF94 ^2^	ORF125	VR14/15	VR23/24
S1	Farm	*P. monodon*	April 2012	4	3	del	7	5950	10,971
S2		*P. monodon*	September 2012	2	3	del	6	5950	10,971
		*P. monodon*	December 2014	2	3	del	6	5950	10,971
		*P. monodon*	May 2015	1	3	del	5	5950	10,971
		*F. indicus*	November 2014	4	3	del	5	5950	10,971
		*F. indicus*	November 2014	2	3	del	6	5950	10,971
S3		*P. monodon*	October 2012	1	3	del	6	5950	10,971
		*F. indicus*	October 2012	2	3	del	6	5950	10,971
Narinda bay	Wild	*F. indicus*	December 2016	1	3	del	6	5950	10,971
		*M. monoceros*	December 2016	1	3	del	6	5950	10,971
Mahajamba bay		*P. monodon*	December 2016	1	3	del	6	5950	10,971
		*M. monoceros*	December 2016	1	3	del	6	5950	10,971
Tsiribihina estuary		*F. indicus*	August 2016	8	3	del	6	5950	10,971
		*F. indicus*	August 2016	4	3	del	8	5950	10,971
		*M. monoceros*	August 2016	3	3	del	6	5950	10,971
		*M. japonicus*	August 2016	1	3	del	6	5950	10,971
		*M. rosenbergii*	August 2016	2	3	del	6	5950	10,971

^1^*P monodon: Penaeus monodon; F. indicus: Fenneropenaeus indicus; M. monoceros: Metapenaeus monoceros; M. japonicus: Marsupenaeus japonicus; M. rosenbergii: Macrobachium rosenbergii.*^2^ del: deletion. ORF: Open Reading Frame; VR: Variable Region.

## Supplementary Materials

The following are available online at https://www.mdpi.com/article/10.3390/v13091713/s1, Table S1: GenBank Accession no. of DNA sequences used in this study.
