# Supplementary material for: Genotype Diversity and Spread of White Spot Syndrome Virus (WSSV) in Madagascar (2012–2016)
Source: Viruses. 2021 Aug 28;13(9):1713. doi: 10.3390/v13091713 (PMC8472404; doi:10.3390/v13091713)
Supplement: Supplementary file 1 [file viruses-13-01713-s001.zip › viruses-1285378-supplementary.pdf]

Supplementary Table S1: GenBank Accession number of variable loci (ORF125, ORF73/77, ORF93/96, VR14/15, VR23/24) DNA sequences of *white spot syndrome virus* (WSSV) strains isolated in Madagascar.

**Variable loci**	**Strain**	**Length (bp)**	**GenBank Accession**
ORF125	WSSV-MD-I-2012	721	MZ327619
ORF125	WSSV-MD-II-2012	652	MZ327620
ORF125	WSSV-MD-III-2014	583	MZ327621
ORF125	WSSV-MD-IV-2016	792	MZ327622
ORF93-ORF96	WSSV-MD-I-2012	348	MZ327623
ORF73-ORF77	WSSV-MD-I-2012	1739	MZ327624
VR14/15	WSSV-MD-I-2012	1850	MZ327625
VR23/24	WSSV-MD-I-2012	1265	MZ327626
